# Effect of Clinoptilolite and Halloysite Addition on Biogas Production and Microbial Community Structure during Anaerobic Digestion

**DOI:** 10.3390/ma13184127

**Published:** 2020-09-17

**Authors:** Martyna Ciezkowska, Tomasz Bajda, Przemyslaw Decewicz, Lukasz Dziewit, Lukasz Drewniak

**Affiliations:** 1 Department of Environmental Microbiology and Biotechnology, Faculty of Biology, Institute of Microbiology, University of Warsaw, Miecznikowa 1, 02-096 Warsaw, Poland; mciezkowska@gmail.com (M.C.); decewicz@biol.uw.edu.pl (P.D.); ldziewit@biol.uw.edu.pl (L.D.); 2 Department of Mineralogy, Petrography and Geochemistry, Faculty of Geology, Geophysics and Environmental Protection, AGH University of Science and Technology, Mickiewicza 30, 30-059 Krakow, Poland; bajda@agh.edu.pl

**Keywords:** zeolite, halloysite, anaerobic digestion, biogas, microbial community, microorganisms immobilization, maize silage, sewage sludge

## Abstract

The study presents a comparison of the influence of a clinoptilolite-rich rock—zeolite (commonly used for improving anaerobic digestion processes)—and a highly porous clay mineral, halloysite (mainly used for gas purification), on the biogas production process. Batch experiments showed that the addition of each mineral increased the efficiency of mesophilic anaerobic digestion of both sewage sludge and maize silage. However, halloysite generated 15% higher biogas production during maize silage transformation. Halloysite also contributed to a much higher reduction of chemical oxygen demand for both substrates (by ~8% for maize silage and ~14% for sewage sludge) and a higher reduction of volatile solids and total ammonia for maize silage (by ~8% and ~4%, respectively). Metagenomic analysis of the microbial community structure showed that the addition of both mineral sorbents influenced the presence of key members of archaea and bacteria occurring in a well-operated biogas reactor. The significant difference between zeolite and halloysite is that the latter promoted the immobilization of key methanogenic archaea *Methanolinea* (belong to *Methanomicrobia* class). Based on this result, we postulate that halloysite could be useful not only as a sorbent for (bio)gas treatment methodologies but also as an agent for improving biogas production.

## 1. Introduction

Ensuring highly efficient biogas production from the anaerobic digestion (AD) of biomass is strictly related to the control of various operational parameters (e.g., pH, temperature, hydraulic retention time, ammonia concentration), which directly or indirectly influence the activity of microorganisms involved in the process [1]. One of the treatment methods used to improve biogas production is the addition of various mineral sorbents, which boost the activity of microorganisms involved in the anaerobic digestion of various substrates [2,3]. Most sorbents are raw materials of natural origin and are used in a powder or granular form. The sorbents are porous materials characterized by their ability to: (i) lose and gain water reversibly, (ii) adsorb molecules with an appropriate cross-sectional diameter, and (iii) exchange their constituent cations without a major change in their structure [4]. Mineral sorbents’ porous structure and their ability to exchange constituent cations allow for the reduction of toxicity in the environment (e.g., the reduction of ammonia and ammonium ion concentrations in the solution) [5], supplementation (e.g., Ca^2+^ and Mg^2+^ ions released from zeolite) [6], and immobilization of microbial cells (e.g., high capacity for colonization of microorganisms on their surface) [7]. The most commonly described mineral sorbents used to enhance the anaerobic digestion process are zeolites [8].

Zeolites are crystalline-hydrated aluminosilicates, which contain SiO_2_, Al_2_O_3_, Fe_2_O_3_, MgO, CaO, Na_2_O, and K_2_O. There are about 50 different types of zeolite with various mineralogical compositions depending on their structure and Si/Al ratio [9,10]. The properties of zeolites are useful in improving the anaerobic digestion process of feedstock with high concentrations of nitrogen compounds, such as cattle, pig and chicken wastes, food wastes, slaughterhouse wastes, or sewage sludge as they prevent process inhibition [5,11,12,13]. Firstly, zeolites are used as an ion exchanger for ammonia removal in anaerobic digestion. Also, this property can be useful for removing toxic materials (such as sulphur or phenolic compounds) that can inhibit the growth of microorganisms responsible for the anaerobic digestion process [14]. Moreover, the ion exchange can also be used to activate the mineral sorbent with trace metals (Fe, Co, Mo, Ni, Cu or Zn) that are essential for microbial enzymatic activities [15].

Zeolite can enhance biogas production by the immobilization of microbial cells on their surface, which leads to higher microbial activity. The ability of microorganisms to colonize zeolite involved in biogas production using grass silage [16], swine manure [17], and vinasses [18] as substrates was confirmed by using scanning electron microscopy visualization. Moreover, changes in the biogas community using 16S rRNA gene profiling were analyzed and described in a few reports [6,19,20]. Interestingly, Ziganshina and colleagues [17] showed that the presence of zeolite impacts biogas production efficiency but not microbial community structure. By contrast, Lin and colleagues [3] showed that the addition of zeolite influenced both biogas production efficiency and microbial community structure. Since a literature search delivered ambiguous results, in this study, we re-examined the influence of zeolites on the composition of the microbial community during anaerobic digestion. This is crucial as biological factors are obligatory during biogas production, and microbial composition can be influenced by many factors, like substrate type, the origin of microorganisms, organic load, etc.

Despite extant knowledge of the application of zeolites in anaerobic digestion and biogas production, little is known about other minerals that may enhance the process. One of them is halloysite—a unique tubular nanoclay, which has recently attracted a growing scientific interest [21]. Halloysite is a 1:1 aluminosilicate clay mineral with the empirical formula Al_2_Si_2_O_5_(OH)_4_ [22]. It was formed naturally millions of years ago, and now substantial halloysite deposits are available in numerous countries [23]. Similar to other mineral sorbents, halloysite has a porous structure, which may also be used to immobilize microorganisms (similarly to zeolite) during the anaerobic digestion process. It offers unique properties, including a high surface area and the ability to promote microorganism adhesion and ion exchange reactions [24]. Halloysite naturally occurs as small cylinders (nanotubes). Owing to the layered structure of the halloysite, it has a large specific surface area, range from 22.1 to 81.6 m^2^/g [25] (to compare, zeolite has average surface area 24.9 m^2^/g) [4]. In addition, halloysite is inexpensive, and the world supply is in excess of 1000 tons per year (average of 4 dollars per one kilogram) [26]. Therefore, the main aim of this study was to compare the effect of the addition of halloysite and a well-known clinoptilolite-rich rock, zeolite, on biogas production from sewage sludge or maize silage.

## 2. Materials and Methods

### 2.1. Inoculum and Substrate

Inocula were taken from: (i) a fermenter tank of an agricultural biogas plant in Miedzyrzec Podlaski (Poland), fed with maize silage and operated at 37 °C temperature, or (ii) sewage sludge from a fermentative tank from the municipal sewage treatment plant in Lodz (Poland), operated at 37 °C temperature. Methanogenic inocula were sampled in a hermetic canister or container, transported to the laboratory, and stored for a maximum of 16 h at a temperature of 37 °C.

In experiments, the bioreactors were fed with maize silage provided by a farm located in Mikanow (Poland) or sewage sludge from the municipal wastewater treatment plant in Lodz (Poland). A bulk amount of maize silage was transported from Mikanow to the laboratory at room temperature, portioned into plastic bags, and stored at 4 °C. The sewage sludge was sampled in a canister or container, transported to the laboratory, and stored at 4 °C. The physico-chemical characteristics of the methanogenic inocula and substrate are shown in Table 1.

### 2.2. Minerals

The clinoptilolite-rich rock (zeolite) was sourced from the Sokyrnytsya deposit (Trans Carpathian region, Ukraine) [27], and the halloysite sample (H) was taken from Dunino, Poland [28]. The most important parameters that determine the textural, structural, and sorption properties of zeolite and halloysite, as well as their chemical and mineral composition, are presented in Table 2. The zeolite used in the study was rich in clinoptilolite (~75%). K-feldspar and quartz were identified as admixtures [29]. The halloysite sample contained ~60% halloysite and ~40% kaolinite [28].

The chemical composition of the investigated samples was determined with a sequential wavelength dispersive X-ray fluorescence spectrometer (WDXRF) RIGAKU ZSX Primus II with Rh anode (4.0 kW) (RIGAKU, Tokyo, Japan). The samples were ground to a particle size of 200-mesh and then calcined at 1000 °C. The calcined samples were homogenized with flux (Li_2_B_4_O_7_) at a ratio of 1:10 and melted at 1050 °C until a glass pastille with a diameter of 25 mm was obtained. This system allowed for multi-elemental detection of elements from boron to uranium. The analyses were conducted in combustion conditions. The cation exchange capacity (CEC) of the samples was determined on the basis of the amount of Ba^2+^ ions saturated in the sample and desorbed by 1M MgCl_2_ [30]. The specific surface area and porosity were determined as described earlier [31]. Firstly, the samples were outgassed for 12 h at 105 °C. Secondly, N_2_ gas adsorption/desorption isotherms at −196 °C were performed using an ASAP 2020 apparatus (Micromeritics, Norcross, GA, USA). The specific surface area (*S_BET_*) was calculated based on the data obtained from N_2_ isotherms, by using the Brunauer–Emmett–Teller (BET) equation [32]. The volume of total pore was calculated from the amount of N_2_ adsorbed at a relative vapor pressure (P/P0) of approximately 0.99. The Dubinin–Radushkevich method was used to calculate the micropores volume (Vmic) [33]. The Barrett–Joyner–Halenda (BJH) method was used to determine the volume of mesopore (Vmez) [34] in the mesopore range described by Dubinin [33]. The volume of macropore (Vmac) was calculated by using the following Equation:

(1) $$V_{mac}=V_{tot}^{0.99}-\left(V_{mic}^{DR}+V_{mes}^{BJH}\right)$$

where $V_{mic}^{DR}$ is the volume of micropores, and $V_{mes}^{BJH}$ is the volume of mesopores.

### 2.3. Experimental Design

The experiments were carried out in laboratory batch-reactors with a working volume of 800 cm^3^, made of 1 dm^3^ GL 45 glass bottles (Schott Duran, Wertheim, Germany) connected with Dreschel scrubbers and 1 dm^3^ Tedlar gas bags (Sigma-Aldrich, St. Louis, MO, USA) as a biogas collector. Biomethanization of organic matter lasted for 30 days and was performed at 37 °C. The lab-scale bioreactors were supplied with: (i) inoculum–fermentative sludge from agricultural biogas plant or fermentative sludge from the municipal sewage treatment plant at a concentration of 10 g VS/dm^3^; (ii) feedstock–maize silage or sludge sewage from the municipal sewage treatment plant at a concentration of 10 g VS/dm^3^; and (iii) drinking spring water was added to make up the 800 cm^3^ total volume. The doses of minerals used in the experiments were 5 g/dm^3^. The control variants were not supplemented with minerals. Each experiment was carried out in triplicate.

### 2.4. Analytical Methods

To control the anaerobic digestion process, the following parameters were determined: volume and methane content in the biogas, volatile fatty acids (VFAs), total solids (TS), volatile solids (VS), chemical oxygen demand (COD), total ammonia nitrogen (TAN), and pH. Total solids and volatile solids analyses were performed according to standard methods described in the American Public Health Association Standard Methods [35]. Volatile fatty acids, chemical oxygen demand, and total ammonia nitrogen were determined using Nanocolor^®^ kits (Machery-Nagel GmbH, Duren, Germany). Biogas production was monitored daily with a MilliGascounter MGC-1 (Ritter, Bochum, Germany). Methane content was analyzed by a gas analyzer DP-28 (Nanosens, Tarnowo Podgorne, Poland). Significant differences (*p* < 0.05) in the biogas production and physico-chemical parameters in the variants of the experiments were evaluated by using a *t*-test.

### 2.5. DNA Extraction and 16S rRNA Gene Amplification

The metagenomic DNA was extracted in triplicate by using a DNA extraction kit for soil samples, the PowerSoil DNA Extraction Kit (MoBio Laboratories, Carlsbad, CA, USA) following the manufacturer’s recommendations. Free microorganisms were extracted from 0.5 g of sludge samples from each inoculation phase. To collect mineral-immobilized microorganisms, 0.5 g of mineral sorbent was rinsed three times with 500 μL of 1× phosphate-buffered saline (PBS, pH 8.0) before applying the DNA extraction procedure. The concentration and quality of extracted DNA were estimated using the NanoDrop 2000 instrument (Thermo Scientific, Waltham, MA, USA) and DNA electrophoresis. The metagenomic DNA was used for amplicon preparation in the polymerase chain reaction (PCR) reaction with the following modified primers: Arch349F: 5′-TCGTCGGCAGCGTCAGATGTGTATAAGAGACAGGYGCASCAGKCGMGAAW-3′ and Arch806R: 5′-GTCTCGTGGGCTCGGAGATGTGTATAAGAGACAGGGACTACVSGGGTATCTAAT-3′ targeting the variable region V3 and V4 of an archaeal 16S rRNA gene and, 16S_V3-F: 5′-TCGTCGGCAGCGTCAGATGTGTATAAGAGACAGCCTACGGGNGGCWGCAG-3′ [36] and 16S_V4-R (modified Bakt_341F and Bakt_805R): 5′-GTCTCGTGGGCTCGGAGATGTGTATAAGAGACAGGACTACHVGGGTATCTAATCC-3′ [37,38] targeting the variable regions V3 and V4 of a bacterial 16S rRNA gene. The reaction mixture (50 µL) contained 100 ng of template DNA and primers and 0.02 U of KAPA HiFi polymerase (KAPA Biosystems, Sigma-Aldrich, St. Louis, MO, USA).

Archaeal and bacterial 16S rRNA fragments were PCR-amplified in a Mastercycler Nexus GX2 thermocycler (Eppendorf, Hamburg, Germany) with 30 and 25 cycles, respectively. PCR conditions were as follows: initial denaturation (3 min at 95 °C); 30 or 25 cycles composed of denaturation (30 s at 95 °C), annealing (30 s at 71 °C for archaea and 72 °C for bacteria), extension (30 s at 72 °C); and a final extension (5 min at 72 °C). The PCR products were analyzed by DNA electrophoresis in 2% agarose gels. Each PCR reaction was repeated in triplicate, and then every three probes were mixed, and the mixture was used for the sequencing.

### 2.6. Metagenomic Library Preparation and Amplicon Sequencing

To prepare libraries, approximately 250 ng of amplified DNA (pooled from the three PCR replicates) was used with the Illumina TruSeq DNA Sample Preparation Kit (Thermo Scientific, Waltham, MA, USA) according to the manufacturer’s protocol. Libraries were verified using the 2100 Bioanalyzer (Agilent, Santa Clara, CA, USA) High-Sensitivity DNA Assay and KAPA Library Quantification Kits (Thermo Scientific, Waltham, MA, USA).

The amplicon libraries were sequenced using an Illumina MiSeq instrument (Illumina, San Diego, CA, USA) in the DNA Sequencing and Oligonucleotide Synthesis Laboratory (oligo.pl) at the Institute of Biochemistry and Biophysics, Polish Academy of Sciences, with the use of a v3 MiSeq chemistry kit in the paired-end mode.

### 2.7. Bioinformatics Analysis

For archaeal and bacterial diversity analyses, raw Illumina MiSeq pair-end reads obtained from the sequencing of V3 and V4 regions of 16S rRNA genes were processed separately, using mostly tools and pipelines wrapped by Quantitative Insights Into Microbial Ecology (QIIME) [39]. At first, read quality and length filtering was performed with CutAdapt v1.9.1 [40]; for instance, adapters and low-quality nucleotides (<Q20) were trimmed, and reads longer than 150 nucleotides were assembled using SeqPrep v1.1 (https://github.com/jstjohn/SeqPrep) with a minimum 15 base overlap and 90% identity in the overlapping region. The resulting sequences were filtered by length (400–500 nt were kept) and checked for chimeras with VSEARCH v2.4.4 [41] https://www.ncbi.nlm.nih.gov/pmc/articles/PMC5075697/), using both de novo and reference-based methods. During the reference-based chimera search, the SILVA QIIME release 128 nr 97 dataset was used as the reference [42]. Sequences classified as chimeras were removed from further analysis. Afterwards, reads were clustered into Operational Taxonomic Units OTUs at 97% identity within centroids and with a minimum number of three sequences per OTU with USEARCH v6.1 (https://www.drive5.com/usearch/), following the open-reference strategy implemented in QIIME. The taxonomy was assigned using an RDP classifier (https://rdp.cme.msu.edu/classifier/classifier.jsp) v2.2 [43], with the confidence threshold set to 0.8 using the SILVA QIIME release 128 nr 97 reference dataset.

## 3. Results and Discussion

### 3.1. Biogas Production Enhancement by Mineral Sorbent Addition

**Figure 1:**
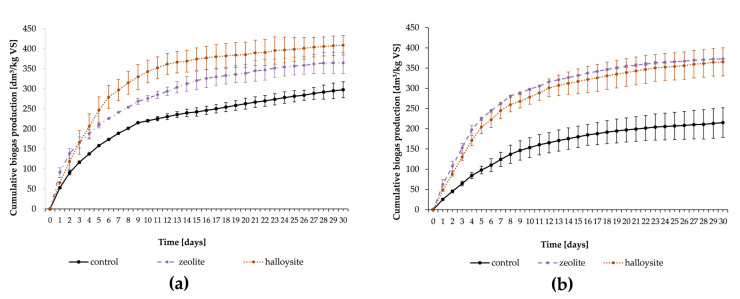
Cumulative biogas production during batch anaerobic digestion of maize silage (**a**) and sludge sewage (**b**) with or without minerals (control). Error bars represent the standard deviation of triplicate measurements.

Halloysite and zeolite were added to batch anaerobic digestion at the final concentration of 5 g/dm^3^. Figure 1 shows cumulative biogas production during the anaerobic digestion of maize silage (Figure 1a) and sewage sludge (Figure 1b) with or without the addition of tested mineral sorbents. For both substrates, the addition of minerals had a positive effect on biogas production. Cumulative biogas production after 30 days of anaerobic digestion of maize silage without minerals (control) was 293 ± 20 dm^3^/kg VS, whereas in cultures with halloysite and zeolite, cumulative biogas production was 409 ± 24 and 365 ± 26 dm^3^/kg VS, respectively (Figure 1a). Addition of these minerals to maize silage anaerobic digestion resulted in enhanced biogas production (up to 37% for halloysite and 22% for zeolite). In turn, cumulative biogas production in the anaerobic digestion of sewage sludge without the addition of minerals (control) was 215 ± 37 dm^3^/kg VS, whereas in cultures with halloysite and zeolite, it was 365 ± 37 and 373 ± 0.4 dm^3^/kg VS, respectively (Figure 1b). The addition of the minerals in this case resulted in statistically significant enhanced biogas production of up to 70% for halloysite and up to 73% for zeolite.

The addition of zeolites significantly increased biogas production, but the efficiency was similar to the results achieved in other papers. In this study, the addition of 5 g/dm^3^ of zeolite increased biogas production from sewage sludge by up to 70% and from maize silage by up to 22%. In other papers, biogas yield was increased by 67% with the addition of 10 g/dm^3^ of zeolite in the batch anaerobic digestion of swine manure [44]. Moreover, the addition of 2–4 g/dm^3^ of zeolite in the batch anaerobic digestion of swine manure increased methane yield by 80–100% [14]. In another paper, Tao et al. [5] demonstrated that CH_4_ yields were improved by 54% during the anaerobic digestion of sewage sludge with zeolites. According to Kougias and colleagues [45], zeolite at doses of 10 g/dm^3^ in batch treatments increased methane production during the mesophilic anaerobic decomposition of raw poultry manure by up to 110%.

According to the results of this work, halloysite appears to be an effective mineral sorbent for enhancing biogas production from both substrates: sewage sludge and maize silage. In the experiment with sewage sludge, halloysite increased biogas production at a similar level to zeolite (halloysite 70%, zeolite 73%). Meanwhile, in the batch anaerobic digestion of maize silage, halloysite produced a much greater biogas yield (halloysite-up to 37%, zeolite 22%). This effect may be related to the fact that halloysite has a greater proportion of macropores (Vmac 30%) than zeolite (Vmac 24%). Macropores have sufficient size to provide a livable space environment for bacteria. Macropore cavities size are larger than 50 nm, mesopores are between about 2 and 50 nm, and micropores are less than 2 nm [46]. The presence of macropores mainly lead to has a larger specific surface area (halloysite 49.4 m^2^/g and zeolite 30.3 m^2^/g), which may enhance the colonization of microorganisms involved in anaerobic digestion. The bacterial biofilm attached on the minerals’ surface is less prone to washout compared with the suspended microorganism in the feedstock solution [7]. Notwithstanding, the findings of this paper require further investigation, such as analyzing the microbial community on the mineral surface.

Despite the increased biogas yield, we did not find that the biogas produced by the anaerobic digestion of maize silage and sewage sludge with the addition of both tested mineral sorbents was of superior quality. In this experiment the biogas quality is determined by methane content in the biogas produced. The methane concentration remained the same, and no significant differences in methane content were observed among the treatments. The maximal concentration of methane in produced biogas during anaerobic digestion from maize silage in the control variant was 72%. Meanwhile, in batch anaerobic digestion with the tested minerals, zeolite and halloysite, the methane concentrations of the biogas produced were 72% and 71%, respectively. In anaerobic digestion from sewage sludge, there was also no significant difference in the methane concentration of the produced biogas. The maximal methane concentration of the biogas in the control variant produced by the anaerobic digestion of sewage sludge was 67%, whereas, in tested variants, it was 68% (zeolite) and 66% (halloysite). The observed effect of the addition of mineral sorbents on the quality of biogas is consistent with the current literature. Kotsopoulos and colleagues [47] also showed that the addition of zeolites in the anaerobic digestion of pig waste enhanced biogas production but did not increase methane concentration in the produced biogas.

### 3.2. Effects of Mineral Sorbents on the Biodegradation of Organic Matter

Biodegradable organic substances can be removed through conversion into CH_4_ and CO_2_ during the anaerobic digestion process, resulting in a decrease in COD and volatile solids. The addition of zeolite and halloysite increased the rate of reduction of COD from 21% to 32% and 41% in the experiment with maize silage and from 36% to 52% and 65% in the experiment with sewage sludge, respectively (Table 3). The highest reductions of COD in both experiments were achieved in anaerobic digestion with halloysite. The addition of halloysite increased the reduction of COD by up to two-fold in comparison with the controls (Table 3).

The reduction of volatile solids also increased, from 45% to 51% and 48% in the experiment with sewage sludge after supplementation with zeolite and halloysite and from 32% to 45% and 54% in the experiment with maize silage, respectively. The reduction efficiency of VS was statistically significantly higher in treatments with halloysite compared to the anaerobic digestion of maize silage without minerals. During the anaerobic digestion of sewage sludge, the addition of zeolite and halloysite had the same influence on the reduction of organic matter.

The removal efficiency of TS was also higher in anaerobic digestion with minerals, remaining at a similar level for both substrates. The reduction increased from 41% to 44% and 45% in the experiment with sewage sludge supplemented with zeolite and halloysite and from 20.15% to 24% and 29% in the experiment with maize silage, respectively.

In the variant with maize silage as substrate, the addition of halloysite resulted in a higher reduction of VS than the addition of zeolite, and this also corresponded to the results for biogas production and COD reduction. Biogas production increased by up to 37% (halloysite) and 22% (zeolite), and COD was reduced by up to 41% and 32%, respectively. In the variant with sewage sludge, the results for VS reduction also corresponded with the results for biogas production. Biogas production and VS reduction during the anaerobic digestion of sewage sludge for both minerals were statistically similar, biogas yield increased by up to 70% (halloysite) and 73% (zeolite). VS reduction was also significant—that is, 48% (halloysite) and 51% (zeolite)—compared with the control (45%).

The possible explanation for reduction of organic matter in the both variants is that minerals could promote the microorganisms growth, which is helpful in the effective degradation of insoluble macro-molecular organic compounds of substrates, and enhanced biogas production [48]. Moreover, the higher COD reduction in the variants with halloysite for both substrates could be associated with the porous structure of halloysite. Such structure is conducive to adsorb the small molecule organic matters in anaerobic digestion system [49]. An additional argument for halloysite is its larger specific surface area and a greater proportion of macropores than zeolite.

The enhanced reduction of VS as a consequence of zeolite addition was reported previously. Wijesinghe et al. [50] indicated that the addition of zeolite increased VS reduction from 41% to 55%. Kotsopoulos et al. [47] showed that the addition of mineral sorbents (zeolites) could also increase VS reduction (in the range of 34 to 49%) in the anaerobic digestion of swine manure. COD removal in batch anaerobic digestion with zeolite has also been reported previously. Purnomo et al. [7] showed that during batch anaerobic digestion of maize silage and cutlery manure, COD concentration decreased by up to 34% compared to the control. Meanwhile, Wang et al. [51] reported that the addition of zeolite resulted in COD removal of up to 75% in anaerobic digestion of organic wastes. It is worth to mention that the reduction of COD is also depends on the substrates which was used for anaerobic digestion. Also, the diameter of used zeolite in anaerobic digestion has influence on COD removal [8].

### 3.3. Effects of Mineral Sorbents on Total Ammonia Nitrogen Concentration

The total ammonia nitrogen concentrations in both variants of the experiment are presented in Table 4. In the controls (with no additive) of anaerobic digestion form sewage sludge or maize silage, TAN concentrations were at the high level (1000.0 mg/dm^3^ and 1897.5 mg/dm^3^, respectively). The addition of zeolite and halloysite contributed to the decrease in TAN concentration during the anaerobic digestion of sewage sludge to 860.0 mg/dm^3^ and 820.0 mg/dm^3^, respectively. However, concentrations of TAN during the anaerobic digestion of maize silage with zeolite and halloysite were statistically unchanged at 1820.0 mg/dm^3^ and 1975.0 mg/dm^3^, respectively.

Ammonium nitrogen is an essential agent for bacterial growth, but at excessive concentrations, it also inhibits the anaerobic digestion process. It was reported that the anaerobic digestion process is inhibited at any operating pH when TAN exceeds 4000 mg/dm^3^ [52]. A high ammonia concentration may explain the limited biogas yield in both control anaerobic digestion experiments. In this paper, TAN concentrations were decreased during the anaerobic digestion of sewage sludge by up to 14% and 18% for zeolite and halloysite, respectively. In previous studies, it was also reported that the addition of clay minerals in anaerobic digestion reduced the toxic effect of ammonia by the minerals’ ability to remove toxic TAN via cation exchange and adsorption into the pores, which resulted in increased methane production [5,12,50,53]. On the other hand, Tada et al. [49] showed that the addition of mineral sorbents resulted in significant NH_4_^+^ removal from natural organic sludge, but methane production was not enhanced. Another paper showed that a reduction in TAN was not achieved during anaerobic digestion with zeolite. In this case, the authors postulated that the attachment of microorganisms to the surface and incorporation inside the zeolite as the operational environment could support higher microbial activity, resulting in very high doses of TAN [17].

In an experiment using maize silage as the substrate for biogas production, there were no statistically significant differences in TAN concentrations in culture with the addition of minerals sorbent in relation to the control (Table 4). In the anaerobic digestion of maize silage, the concentration of TAN in the control was 1897.5 mg/dm^3^ (much higher than in the experiment with sewage sludge). The high concentration of TAN in the anaerobic digestion of maize silage was a consequence of using swine manure as a co-substrate in the biogas plant, which acted as a source of maize silage. The lack of reduction of TAN may be the consequence of its initial high concentration and the low cation exchange capacity of the sorbents used. To reduce the concentration of TAN, it is necessary to use higher amounts of minerals. As other authors reported, the experiment using the highest dosage (20 g/dm^3^) of zeolite produced the best performance, suggesting that an even higher dosage would be required to deal with substrates with ammonium levels higher than 1740 mg/dm^3^ [13]. However, the enhanced biogas production without the reduction in TAN concentration could be explained by the fact that clay minerals may improve anaerobic digestion not only through the adsorption of ammonium ions. Wang et al. [51] showed that improvements to anaerobic digestion could also be attributed to the adsorption of Ca^2+^, Mg^2+^, which enhance the microbial utilization of these cations during the anaerobic digitation process. Another possible explanation could be that mineral sorbents offer a high-capacity immobilization matrix for microorganisms and a higher cell concentration inside the reactor that improves efficiency [7].

### 3.4. Effects of Mineral Sorbents on Volatile Fatty Acid Concentration

The performance of volatile fatty acids during the anaerobic digestion of maize silage and sewage sludge is presented in Table 4. The final concentration of VFAs (after 30 days of batch culture) during anaerobic digestion with tested minerals was lower than in control variants. The addition of minerals contributed to the statistically significant decrease of VFA concentration from 2156.5 mg/dm^3^ to 2100.0 mg/dm^3^ (for zeolite) and 1850.0 mg/dm^3^ (for halloysite) in the experiment with maize silage was observed to have decreased from 172.0 mg/dm^3^ to 155.3 mg/dm^3^ (for zeolite) and 148.7 mg/dm^3^ (for halloysite) in the experiment with sewage sludge, but not statistically significant (*p* < 0.086). It is important to note that the high VFA concentration in cultures with maize silage is a consequence of a higher content of insoluble organic matter (Table 1). Therefore, the presence of zeolite and halloysite promotes the consumption of VFAs during the biogas production process, as for both substrates decreased (between 7% and 14%) VFA concentrations were observed.

Volatile fatty acids are the important substrates in the acetoclastic methanogenesis, so they play a role in promoting methane generation during the anaerobic digestion process [54]. On the other hand, the high concentration of VFA leads to low pH value which may inhibit methanogens [55]. Also VFAs themselves, particularly their dissociated form, can cause inhibition [48]. An increase in concentrations of VFA could be observed during the first days of digestion when the insoluble macro-molecular organic compounds were degraded. However, after 15 days of increased VFA concentrations, biogas production might be inhibited, weakening methanogenesis [56]. Thus, a suitable balance between the rates of hydrolysis and methanogenesis is essential for higher methane production. Rapid methanogenesis is required to prevent accumulation of VFAs lowering pH to an extent that inhibits methanogenesis [3]. Moreover methanogenesis could also be inhibited by other factors such as TAN concentration [6].

Lin et al. [6] showed that zeolite addition significantly increased VFA consumption during the anaerobic digestion process. In this study, it was reported that TAN concentration has a more detrimental effect on the growth of propionic acid-degrading acetogenic bacteria than methanogenic organisms. The addition of mineral sorbents reduced TAN concentration. Propionic acid-degrading acetogenic bacteria under a lower ammonium-level consumed more VFAs, and biogas production achieved a higher yield compared with the control.

Based on our study, we may hypothesize that a reduction in TAN concentration and access to various cations released from mineral sorbents can promote acidogenic bacterial growth. Thus, the biogas production in cultures with maize silage were not inhibited despite the high VFA concentration.

### 3.5. Characterization of Microbial Communities during the Anaerobic Digestion of Sewage Sludge

Sewage sludge is used in this experiment as a model substrate in the energy recovery process from organic waste materials. Microbial community compositions were characterized using bacterial and archaeal hypervariable V3–V4 regions of 16S rRNA gene sequencing. The structure of microbial consortia was analyzed: (i) at the beginning of each batch anaerobic digestion experiment (day 0), (ii) at the end of the batch experiment (day 30), and from biofilms formed on the minerals’ surfaces (day 30–colonization).

#### 3.5.1. Effects of Mineral Sorbent Addition on Bacterial Community Structure during Anaerobic Digestion of Sewage Sludge

**Figure 2:**
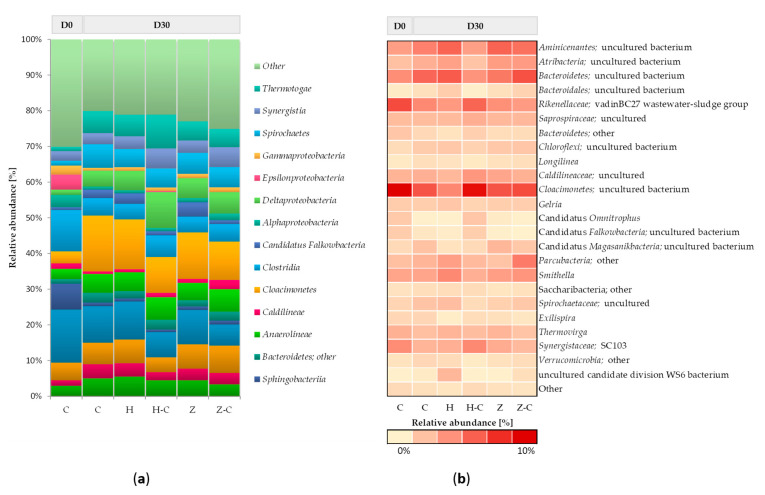
Relative abundance of bacterial OTUs: (**a**) bacterial classes with an abundance > 3% and (**b**) bacterial genera with an abundance > 1% in at least one variant are shown. C: control; H: halloysite; H-C: halloysite-colonisation; Z: zeolite; Z-C: zeolite-colonisation; D0: Day 0; D30: Day 30.

Figure 2 shows the taxonomic composition of bacterial communities in batch anaerobic digestion processes with or without minerals (halloysite or zeolite). At the beginning of the anaerobic digestion process, the bacterial community mainly consists of classes *Bacteroidia*, *Clostridia*, *Sphingobacteriia*, *Bacteroidetes*, and *Epsilonproteobacteria*. After 30 days of anaerobic digestion, changes in the abundance of the dominant classes of bacteria were noticed, and the following classes were recognized in the control (without minerals): *Cloacimonetes*, *Bacteroidia*, *Spirochaetes*, *Bacteroidetes*, *Thermotogae*, *Aminicenantes*, *Anaerolineae*, *Clostridia*, and *Deltaproteobacteria*. No significant differences in bacterial community structure between the control culture and the anaerobic digestion variants with halloysite and zeolite were observed. Only the minor increase of the relative abundance was observed in the classes of *Deltaproteobacteria* (Figure 2a). This result suggested that the addition of mineral sorbents did not disturb the structure of the bacterial community during the anaerobic digestion process.

The most abundant phylum, *Bacteroidetes* (including classes *Bacteroidetes* and *Bacteroidia*), is involved in the hydrolysis and acidogenesis steps of anaerobic digestion [57]. The highly active acidogenic bacteria can consume more VFAs, thus resulting in better biogasification performance. *Bacteroidetes* is mainly responsible for the degradation of complex organic compounds, so the presence of this phylum contributes to the stability of the system with high methane production. Also, bacteria affiliated to *Clostridia*, *Cloacimonetes*, and *Deltaproteobacteria* classes can utilize a broad range of sugars, which is important in the first part of the anaerobic digestion process [58]. Moreover slight increase in the relative abundance of propionate-degrading syntrophs of the genera *Smithella* (belonging to *Deltaproteobacteria*) (Figure 2b) [59]. This result may suggest that those genera can improve degradation of organic matter, which results in increased biogas production in anaerobic digestion process with minerals.

The taxonomical composition of the bacterial community colonizing the material sorbents during anaerobic digestion was also assessed. Both minerals were mostly colonized by bacteria representing the following classes: *Cloacimonetes*; *Deltaproteobacteria*; *Bacteroidia*, *Bacteroidetes*, *Thermotogae*, and Anaerolineae. The comparative analysis of relative abundance of microorganism immobilized on halloysite surface with the community of suspended microorganism in the feedstock solution showed that the relative abundance of *Deltaproteobacteria*, *Thermotogae* and *Clostridia* were increased. Whereas, the abundance of classes *Cloacimonetes* and *Bacteroidia* on halloysite surface was decreased (Figure 2a). On the other hand, increased in the abundance of genera uncultured bacteria belong to class *Cloacimonetes* was observed (Figure 2b).

There was no significant difference between the structure of communities colonizing zeolite and present within the anaerobic batch solution. Also other authors also did not observe the clear evidence of bacteria colonization of zeolite [12].

In this study was demonstrated that halloysite only slightly promote the immobilization of classes: *Deltaproteobacteria*, *Thermotogae* and *Clostridia. Thermotogae* is mainly responsible for the degradation of complex organic compounds [60] and *Deltaproteobacteria* class includes fermenting bacteria species [58] (Figure 2a). Both classes could increase biogas production process. Moreover it is well know that members of *Clostridia* classes are involved in hydrolysis of complex waste materials as well as in acidogenesis and acetogenesis steps. In addition, it was also demonstrated in other studies that bacterial cells of *Clostridium* sp. can be effectively immobilized onto various zeolites and increase the rate of hydrolysis [61].

#### 3.5.2. Effects of Mineral Sorbent Addition on Archaeal Community Structure during the Anaerobic Digestion of Sewage Sludge

**Figure 3:**
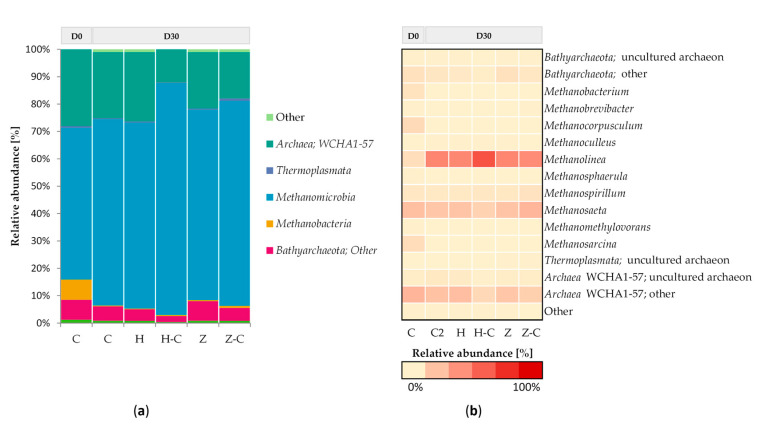
Relative abundance of archaeal OTUs: (**a**) archaeal classes and (**b**) archaeal genera with an abundance > 1% in at least one variant are shown. C: control; H: halloysite; H-C: halloysite-colonisation; Z: zeolite; Z-C: zeolite-colonisation; D0: Day 0; D30: Day 30.

Figure 3 shows the taxonomic composition of archaeal communities in batch anaerobic digestion processes with or without minerals (halloysite or zeolite). At the beginning of the anaerobic digestion process, the archaeal community mainly consisted of Methanomicrobia (including genera of *Methanosaeta* and *Methanolinea*), *Archaea* genera WCHA1-57, *Bathyarchaeota* and *Methanobacterium*. After 30 days of anaerobic digestion with and without minerals, archaeal communities were still dominated by the *Methanomicrobia*, *Archaea* genera WCHA1-57, *Bathyarchaeota*, but *Methanobacterium* was no longer present in the cultures (Figure 3a).

*Methanolinea* belongs to hydrogenotrophic methanogens, which are the key members of a syntrophic propionate-degrading consortia [62]. As other authors reported *Methanosaeta* and *Methanolinea* are well known genera of the Methanomicrobia class able to produce methane [63]. Analysis of archeal 16S rRNA gene sequencing revealed that the addition of mineral sorbents preserved *Methanosaeta* and *Methanolinea* (Figure 3b). Milan et al. [64] showed that the presence of modified natural zeolite favored *Methanosaeta*. In contrast, the other authors showed that the addition of zeolite preserved *Methanosarcina* and enhanced *Methanobacterium* [12].

Analysis of the taxonomic composition of archaeal communities colonizing both minerals revealed that it was similar to the community of suspended microorganism in the feedstock solution. The biofilm community was dominated by *Methanolinea*, *Methanosaeta*, and other genera of *Archaea* WCHA1-57, but the decreased in the abundance of other genera of *Archaea* WCHA1-57 was observed (Figure 3b). *Methanosaeta* slight increased in relative abundance in the archaeal communities immobilized on the zeolites surface compare to the suspended microorganisms in the feedstock solution (zeolite 19%, zeolite colonisation 25%). Fernandez et al. [18] also determined that the anaerobic microorganisms colonized the zeolite is dominated by *Methanosaeta.* The conflicting results concerning the colonization of zeolites were indicated by others authors. Ziganshina et al. [20] showed that various members of genus *Methanobacterium* dominated the archaeal community in the conducted anaerobic digestion, at moderate TAN level, in a fixed zeolite reactor. In the experiment without any inhibitory compound, Weiss et al. [65] determined that zeolite could be colonized by *Methanoculleus*. In the experiments with halloysite, significant difference in the archaeal communities was observed in the abundance of *Methanolinea.* The relative abundance of *Methanolinea* increased form 44% in the suspended microorganisms to 66% in the archaeal communities colonized halloysite surface. This result suggests that halloysite may promote immobilization of *Methanolinea,* members of the family *Methanoregulaceae.* We postulate that halloysite could better enhance immobilization of microorganisms conducting anaerobic digestion process because it has a greater percentage of pores than zeolite. Halloysite has a larger porosity and specific surface area than zeolite (Table 2) and provided a stable environment for growth of attached bacteria in minerals sorbent surface. Forming microorganisms in the biofilms prevent form their wash-out from the systems. Also, higher microbial biomass-attached minerals sorbent increased the activity of microorganisms and improved the efficiency of biogas production process [7].

It is worth noting that increased abundance of *Methanosaeta* or *Methanolinea* in the experiments with zeolite or halloysite, respectively, may be related with decreased abundance of other genera of *Archaea* WCHA1-57 To confirmed the absolute abundance changes it is also essential to show the copy number of 16S rRNA genes.

## 4. Conclusions

The presented study showed that the addition of halloysite as well as zeolites caused a significant increase in the biogas production yield in mesophilic anaerobic digestion of maize silage and sewage sludge. However, halloysite had a much greater impact on maize silage transformation. The batch experiments also confirmed that supplementation by both mineral sorbents directly stimulated COD and VS removal in the anaerobic digestion of both substrates (maize silage and sewage sludge). Furthermore, differences in efficiency between zeolite and halloysite can be correlated with biogas production yield. In turn, the addition of both minerals contributed to a significant reduction in TAN concentration only in the case of anaerobic digestion of sewage sludge. However, during the degradation of maize silage, the effect of reduced ammonia toxicity was not observed, probably because the TAN concentration was too high, and the cation exchange capacity of used sorbents was too low. Analysis of the sequences of 16S rRNA gene amplicons revealed that zeolite and halloysite influenced the changes in the archaeal and bacterial communities. An important difference between the used sorbents is their effect on shifts in the community structures of the immobilized microorganisms. We postulate that halloysite may promote immobilization of *Methanolinea* (belong to *Methanomicrobia* class), a population of key methanogenic archaea. Notwithstanding, this finding requires further investigation, such as quantitative PCR (qPCR) analysis, to show that the copy number of 16S rRNA genes *Methanolinea* is indeed increased.

Based on the results obtained, we can conclude that halloysite is an economically attractive alternative to zeolites to boost the biogas production process from substrates such as maize silage or sewage sludge. However, to evaluate the potential application of halloysite in the anaerobic digestion process it is critically important to determine the effect of halloysite on biogas production stability and changes in the microbial community structure during anaerobic digestion performed in a continuous mode.

## Figures and Tables

**Table 1:** Characteristics of the inoculum and substrate used in the experiment.

Parameters	Units	Inocula			Substrate	
		Sludge from Agricultural Biogas Plant	Sludge from Agricultural Biogas Plant	Maize Silage	Sludge Sewage from Municipal Sewage Treatment Plant
pH	-	7.67	7.53	3.77	5.53
TS *	% FM **	3.6	3.14	37.00	6.73
VS ^†^	% TS	69.91	64.5	96.00	79.34
COD ^††^	g/dm^3^	7.70	0.80	38.90	6.20
VFAs ^#^	g/dm^3^	2.62	0.30	1.05	1.43
TAN ^##^	g/dm^3^	1.23	0.71	not detected	0.31

^††^ COD: chemical oxygen demand; * TS: total solids; ^##^ TAN: total ammonia nitrogen; ^#^ VFAs: volatile fatty acids; ^†^ VS: volatile solid; ** FM: fresh matter.

**Table 2:** Chemical, physical, and mineralogical characteristics of the sample studied.

Parameter	Sample	
	Zeolite	Halloysite
Chemical composition (wt.%)		
SiO_2_	65.24	27.68
TiO_2_	0.18	3.19
Al_2_O_3_	12.58	25.63
Fe_2_O_3_	1.85	21.58
CaO	3.24	1.07
MgO	0.78	0.33
K_2_O	2.88	0.14
Na_2_O	0.64	0.08
LOI *	12.23	18.36
Percent of pores		
Vmic	9.8	7.6
Vmez	66.6	62.3
Vmac	23.6	30.2
Physical–chemical properties and mineral composition		
BET(m^2^/g) **	30.3	49.4
CEC (meq/100 g) ***	142.3	13.5
Mineral composition	Cp ^†^, Fk ^††^, Q ^†††^	H ^#^, K ^##^

* LOI: loss of ignition; ** BET: Brunauer–Emmett–Teller surface area; *** CEC: cation exchange capacity; ^†^ Cp: clinoptilolite; ^††^ Fk: K-feldspars; ^#^ H: halloysite; ^##^ K: kaolinite; ^†††^ Q: quartz.

**Table 3:** Chemical oxygen demand, total solids, and volatile solids reduction percentage (%) achieved at the end of the batch anaerobic digestion process of maize silage and sludge sewage with or without minerals (control).

Parameters (%)	Substrates	Experiments		
		Control	Addition of Zeolite	Addition of Halloysite
COD * reduction	maize silage	21.15 ± 1.92	32.48 ± 1.48	40.60 ± 2.67
	sewage sludge	35.68 ± 5.01	51.55 ± 2.11	65.35 ± 4.90
TS ^#^ reduction	maize silage	20.15 ± 2.46	24.34 ± 0.00	28.88 ± 0.42
	sewage sludge	40.93 ± 0.42	44.46 ± 4.57	44.76 ± 2.91
VS ^†^ reduction	maize silage	32.25 ± 2.94	45.48 ± 0.00	53.58 ± 0.76
	sewage sludge	45.05 ± 2.03	51.26 ± 3.75	48.35 ± 1.19

* COD: chemical oxygen demand; ^#^ TS: total solids; ^†^ VS: volatile solids.

**Table 4:** Final volatile fatty acids and total ammonia nitrogen concentration (mg/dm^3^) in the batch anaerobic digestion process of maize silage and sewage sludge.

Parameters (mg/dm^3^)	Substrate	Experiments		
		Control	Addition of Zeolite	Addition of Halloysite
VFAs *	maize silage	2156.5 ± 17.5	2000.0 ± 99.0	1850.0 ± 106.1
	sewage sludge	172.0 ± 20.0	155.3 ± 7.6	148.7 ± 7.0
TAN ^#^	maize silage	1897.5 ± 82.5	1820.0 ± 77.0	1975.0 ± 124.9
	sewage sludge	1000.0 ± 45.8	860.0 ± 70.0	820.0 ± 113.6

* VFAs: volatile fatty acids; ^#^ TAN: total ammonia nitrogen.

## Abbreviations

AD	Anaerobic Digestion
TS	Total Solids
VS	Volatile Solids
COD	Chemical Oxygen Demand
TAN	Total Ammonia Nitrogen
VFAs	Volatile Fatty Acids
LOI	Loss of Ignition
CEC	Cation exchange capacity
